# A hidden clue behind angioedema in an elderly patient

**DOI:** 10.1093/omcr/omaf157

**Published:** 2025-09-15

**Authors:** Okan Cetin, Ilkim Deniz Toprak, Gamze Kemec, Simge Erdem, Gulcin Yegen, Semra Demir

**Affiliations:** Department of Internal Medicine, Istanbul Faculty of Medicine, Istanbul University, Istanbul, Türkiye; Department of Allergy and Immunology, Istanbul Faculty of Medicine, Istanbul University, Istanbul, Türkiye; Department of Internal Medicine, Istanbul Faculty of Medicine, Istanbul University, Istanbul, Türkiye; Department of Hematology, Istanbul Faculty of Medicine, Istanbul University, Istanbul, Türkiye; Department of Pathology, Istanbul Faculty of Medicine, Istanbul University, Istanbul, Türkiye; Department of Allergy and Immunology, Istanbul Faculty of Medicine, Istanbul University, Istanbul, Türkiye

**Keywords:** acquired angioedema, lymphoma, C1q, rituximab

## Abstract

Acquired angioedema (AAE) is a rare, potentially life-threatening condition caused by bradykinin, typically presenting as recurrent, non-pitting facial swelling without urticaria. Unlike hereditary forms, acquired angioedema arises later in life and may be associated with underlying B-cell lymphoproliferative disorders. We report a 62-year-old woman with persistent antihistamine-unresponsive facial angioedema, fatigue, and moderate splenomegaly, which led to further work-up for AAE. Low C4 and C1 inhibitor (C1-INH) was detected, with normal C1q levels. Given the presence of splenomegaly and cytopenia, further evaluation of lymphoproliferative disease was pursued. Bone marrow biopsy confirmed splenic marginal zone lymphoma (SMZL), and rituximab-based chemotherapy resulted in full remission of both lymphoma and angioedema. This case highlights the importance of evaluating elderly patients with unexplained angioedema and organomegaly for hidden lymphoproliferative disease. Treating the underlying disease can resolve symptoms and improve outcomes.

## Background

Angioedema is a recurrent, self-limiting, and non-pitting swelling of the skin or mucosal tissues that can be mediated by histamine or bradykinin. While histamine-mediated angioedema is often associated with urticaria and responds well to antihistamines or corticosteroids, bradykinin-mediated forms do not. The latter is classified into hereditary angioedema (HAE) and acquired angioedema (AAE). HAE is caused by mutations in the *SERPING1* gene, whereas AAE is typically observed after the age of 40, without a family history and often in association with lymphoproliferative disorders, most notably non-Hodgkin lymphoma (NHL) and monoclonal gammopathies [1, 2].

NHL is a group of cancers affecting the lymphoid system, comprising various subtypes with diverse clinical features. One such subtype is marginal zone lymphoma that includes three variants: extranodal, nodal, and splenic marginal zone lymphoma (SMZL). Among these, SMZL is a rare and slow-growing lymphoma, accounts for 1%–2% of lymphoid cancers and often presents with splenomegaly, fatigue, and cytopenia.

In this report, we describe a patient who’s recurrent, treatment-resistant angioedema ultimately led to the diagnosis of SMZL. Through this case, we aim to highlight the diagnostic challenges posed by AAE and to emphasize the importance of considering underlying malignancies—particularly B-cell lymphoproliferative disorders—in elderly patients presenting with unexplained angioedema.

## Case presentation

A 62-year-old woman presented with a six-month history of episodic facial angioedema, primarily affecting her eyelids and lips. Episodes occurred approximately every two to three weeks, lasted 24–48 h, and were unresponsive to both antihistamines and corticosteroids. There was no associated urticaria, pruritus, or systemic symptoms, such as fever or night sweats; however, she did report progressive fatigue over the past three months.

Her medical history included well-controlled hypertension treated with amlodipine. She had no personal or family history of angioedema. On physical examination, she had mild upper lip swelling and a palpable spleen 4 cm below the left costal margin. Vital signs were stable. Laboratory tests showed normocytic anemia (hemoglobin: 10.2 g/dl; normal range: 12.0–16.0), with normal leukocyte and platelet counts, and elevated C-reactive protein (16 mg/l; normal range: 0–5) and a normal lactate dehydrogenase level. No monoclonal bands were detected on serum electrophoresis. The peripheral blood smear was unremarkable. Viral serologies for HIV, HBV, HCV, EBV, and CMV were negative.

**Figure 1:**
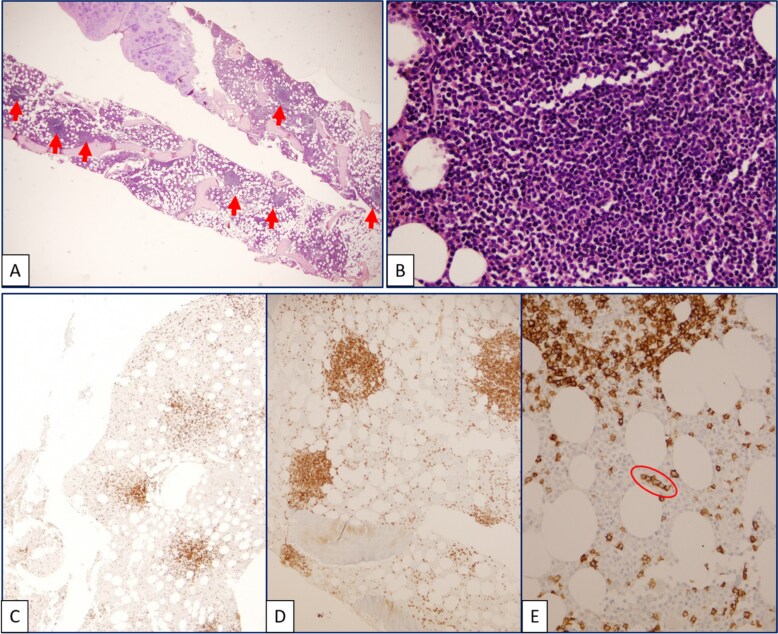
(A) Hypercellular bone marrow biopsy showing interfollicular and focal paratrabecular lymphoid nodules (arrow) (H&E, 2x). (B) Lymphoid cells predominantly consist of small lymphocytes (H&E, 40x). Immunohistochemistry demonstrates that B lymphocytes predominate, highlighted by CD20 (D) compared to CD3 (C). (E) Focal intrasinusoidal infiltration by CD20-positive B lymphocytes is observed (circle).

Given the clinical suspicion of bradykinin-mediated angioedema, complement studies and genetic testing were performed (Table 1). Laboratory findings revealed decreased C4 and C1 inhibitor (C1-INH) levels, along with normal C1q levels. Genetic testing for *SERPING1* was negative, ruling out HAE and confirming the diagnosis of AAE. Treatment plans included the administration of a bradykinin receptor antagonist, icatibant, and plasma-derived C1-INH concentrate under emergency conditions, but neither was required.

**Table 1:** Complement and genetic test results.

**Test**	**Result**	**Reference Range**
C4	1.2 mg/dl	10.0–40.0 mg/dl
C1 inhibitor	0.10 g/l	0.21–0.38
C1q	14.2 mg/dl	11.0–24.0 mg/dl
SERPING1 mutation	Negative	

**Figure 2:**
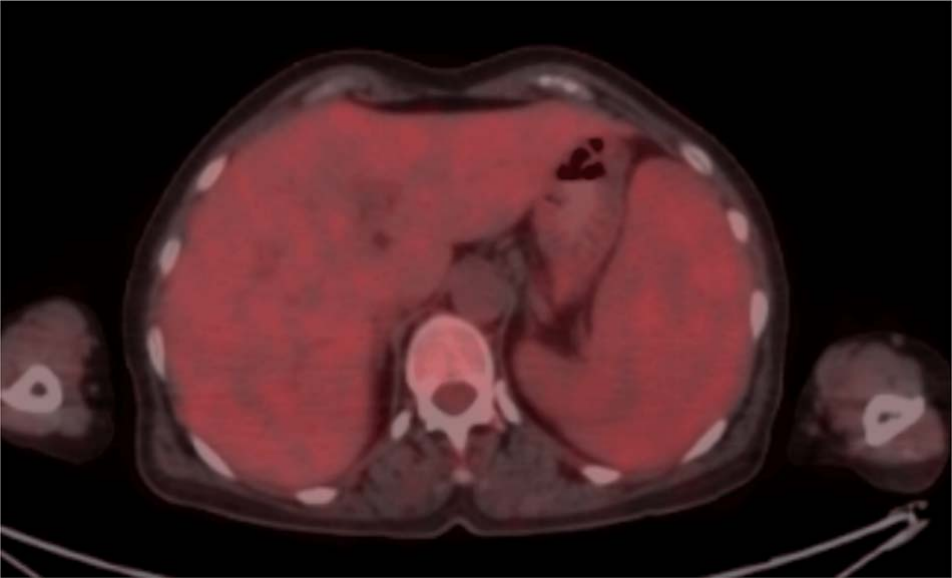
FDG-PET/CT revealed a diffusely mild hypermetabolic appearance in the splenic parenchyma, comparable to the liver.

The presence of unexplained splenomegaly, normocytic anemia, and AAE prompted further investigation for an underlying lymphoproliferative disorder. A bone marrow biopsy was performed based on these clinical clues, in the absence of peripheral smear abnormalities (Fig. 1). Bone marrow histopathology revealed lymphoid nodules containing small lymphocytes, located intertrabecularly and paratrabecularly. Immunohistochemical examination showed a heterogeneous reaction in the lymphoid population with CD3 and CD20, with CD20(+) cells predominating. An intrasinusoidal infiltration pattern was observed with CD20. Additionally, there was a regressive follicular dendritic cell network reaction with CD23 in the center of the nodules. Bcl-1, SOX11, CD10, and Bcl-6 were all negative. CD138 revealed a 5% plasma cell population with a small patch-interstitial distribution. Altogether, these findings were interpreted as being consistent with SMZL. A whole-body FDG-PET/CT scan was performed to evaluate the extent of the SMZL further. The spleen was 16 centimeters in size, and a diffusely mild hypermetabolic appearance was observed in the spleen parenchyma at the same level as the liver (Fig. 2). Additionally, a diffusely mild hypermetabolic appearance was observed in the bone marrow.

Treatment was initiated using a rituximab-based chemotherapy (R-CHOP: rituximab, cyclophosphamide, doxorubicin, vincristine, and prednisone), and significant improvement was observed in the patient’s laboratory and clinical findings after completing six cycles (Table 2). The spleen became non-palpable, and the patient remained free of angioedema episodes following initiation of treatment. Upon completion of therapy, repeat bone marrow biopsy showed no pathological findings.

**Table 2:** The levels of C1 inhibitor and C4 were assessed both before and after the six cycles of R-CHOP (rituximab + cyclophosphamide + doxorubicin + vincristine + prednisone) treatment.

	Before the treatment	After the treatment
C1 inhibitor (0.21–0.38 g/l)	0.10 g/l	0.28 g/l
C4 (10–40 mg/dl)	1.2 mg/dl	15 mg/dl

## Discussion

In this case, AAE presented alongside subtle clinical features, including fatigue, normocytic anemia, and moderate splenomegaly—features that raised suspicion for an underlying lymphoproliferative disorder. This clinical approach led to the correct diagnosis and, ultimately, complete resolution of symptoms following appropriate therapy. Importantly, the presence of unexplained organomegaly and anemia in patients with AAE should prompt clinicians to investigate for lymphoproliferative diseases, as timely recognition may facilitate early diagnosis and effective management.

AAE most often manifests as recurrent episodes of cutaneous and/or mucosal angioedema, typically occurring without urticaria, a clear triggering factor, or a family history of angioedema. The initial diagnostic approach involves measurement of C1-INH and C4 antigen levels [1, 3]. If these tests are low, further investigations—including C1q measurement and genetic testing for *SERPING1* mutations—help distinguish acquired from hereditary forms. While C1q is reduced in approximately 70% of patients with AAE due to activation of the classical complement pathway, it remains normal in HAE [4, 5]. However, as in our patient, C1q can be normal in some cases of AAE, and the reason for this finding is not fully understood. Clinicians should therefore be aware that a normal C1q level does not rule out AAE; clinical context always remains paramount.

Following diagnosis, further evaluation for underlying causes is crucial. The association between AAE and lymphoproliferative disorders—especially B-cell malignancies—has been well-documented, although the precise pathophysiological mechanisms remain unclear. Two leading hypotheses include: production of neutralizing autoantibodies against C1-INH by neoplastic B cells, and excessive consumption of C1-INH secondary to chronic complement activation driven by the lymphoproliferative process [2].

Treatment with rituximab-based chemotherapy was initiated in line with standard guidelines, given the patient’s diagnosis of SMZL accompanied by symptomatic splenomegaly and cytopenia. Treatment of the underlying hematological malignancy with this regimen may also effectively manage AAE, as remission of angioedema flare-ups has been reported following rituximab therapy [6].

In conclusion, AAE served as the presenting complaint in this case, and subtle findings such as fatigue and moderate splenomegaly pointed toward an underlying diagnosis of SMZL. Importantly, normal C1q levels did not exclude the diagnosis of AAE, underscoring the need to interpret laboratory data in the context of the clinical picture. The angioedema resolved entirely following rituximab-based chemotherapy, highlighting that treating the root cause can also control symptoms. For elderly patients with unexplained, persistent angioedema, it’s important to keep B-cell lymphoproliferative disorders in mind, even when more obvious signs of lymphoma are absent.
